# Radiation-Induced Cerebro-Ophthalmic Effects in Humans

**DOI:** 10.3390/life10040041

**Published:** 2020-04-16

**Authors:** Konstantin N. Loganovsky, Donatella Marazziti, Pavlo A. Fedirko, Kostiantyn V. Kuts, Katerina Y. Antypchuk, Iryna V. Perchuk, Tetyana F. Babenko, Tetyana K. Loganovska, Olena O. Kolosynska, George Y. Kreinis, Marina V. Gresko, Sergii V. Masiuk, Federico Mucci, Leonid L. Zdorenko, Alessandra Della Vecchia, Natalia A. Zdanevich, Natalia A. Garkava, Raisa Y. Dorichevska, Zlata L. Vasilenko, Victor I. Kravchenko, Nataliya V. Drosdova

**Affiliations:** 1 National Research Center for Radiation Medicine of the National Academy of Medical Sciences of Ukraine, 53 Illyenko Street, 04050 Kyiv, Ukraine; loganovsky@windowslive.com (K.N.L.); eye-rad@ukr.net (P.A.F.); kvk0906@gmail.com (K.V.K.); psyho2011@gmail.com (K.Y.A.); iperchuk@gmail.com (I.V.P.); valta.eyes@gmail.com (T.F.B.); loganovska.tatiana@gmail.com (T.K.L.); neuroaid@i.ua (O.O.K.); gkreinis@gmail.com (G.Y.K.); mgresko@gmail.com (M.V.G.); masja1979@gmail.com (S.V.M.); zdorenko.leonid@gmail.com (L.L.Z.); zdanevychnataliia@gmail.com (N.A.Z.); Dorichev20@gmail.com (R.Y.D.); vasilenkozlata@ukr.net (Z.L.V.); kvik1105@ukr.net (V.I.K.); ndrozdova@gmail.com (N.V.D.); 2 Dipartimento di Medicina Clinica e Sperimentale Section of Psychiatry, University of Pisa, Via Roma, 67, I 56100 Pisa, Italy; federico.mucci@med.unipi.it (F.M.); alessandradellavecchia@gmail.com (A.D.V.); 3 Dipartimento di Biochimica Biologia Molecolare, University of Siena, 53100 Siena, Italy; 4 Dnipropetrovsk Medical Academy of the Ministry of Health of Ukraine, 9 Vernadsky Street, 49044 Dnipro, Ukraine; garkava@ua.fm

**Keywords:** ionizing radiation, brain, neurocognitive deficits, glaucoma, optic neuropathy, retinopathy, angiopathy

## Abstract

Exposure to ionizing radiation (IR) could affect the human brain and eyes leading to both cognitive and visual impairments. The aim of this paper was to review and analyze the current literature, and to comment on the ensuing findings in the light of our personal contributions in this field. The review was carried out according to the PRISMA guidelines by searching PubMed, Scopus, Embase, PsycINFO and Google Scholar English papers published from January 2000 to January 2020. The results showed that prenatally or childhood-exposed individuals are a particular target group with a higher risk for possible radiation effects and neurodegenerative diseases. In adulthood and medical/interventional radiologists, the most frequent IR-induced ophthalmic effects include cataracts, glaucoma, optic neuropathy, retinopathy and angiopathy, sometimes associated with specific neurocognitive deficits. According to available information that eye alterations may induce or may be associated with brain dysfunctions and vice versa, we propose to label this relationship “eye-brain axis”, as well as to deepen the diagnosis of eye pathologies as early and easily obtainable markers of possible low dose IR-induced brain damage.

## 1. Introduction 

It is well recognized that the human brain and eyes are radiosensitive and radiovulnerable organs. The eye lens is one of the most radiosensitive human tissues, and the retina is at risk for suffering from severe consequences induced by ionizing radiation (IR), such as angiopathy and angiosclerosis [1]. Interests concerning the effects of IR on eye structures are progressively increasing, given the evidence that they are easily accessible, and particularly the retina, which is part of the brain, may represent a reliable indicator of the actual conditions of the central nervous system (CNS). Therefore, it has been proposed that targeting the eye might be useful in designing early detection strategies and the prevention of radiotoxicity not only at its level, but also in the brain [2]. 

Although the notion of peculiar eye lens sensitivity to IR was already circulating for decades (e.g., the first description of IR-induced cataract dates back to the end of the 19th century), only in 2012 did the International Commission on Radiological Protection (ICRP) note how “special attention should be paid to the radiation effects in the lens of the eye and the cardiovascular system”. In addition, taking into account new epidemiological data, the ICRP underlined that some tissue reactions were due to threshold or lower doses than the previous ones [3]. As a result, cataract and circulatory diseases, including those of the retina, were considered as tissue reactions of stochastic nature, with the threshold considered to be 0.5 Gy, irrespective of the rate of dose delivery (i.e., acute, fractionated/protracted or chronic exposure). Although, until now, the mechanisms of cataractogenesis and retina damage are poorly understood, and much less is known about circulatory diseases induced by IR [4], the statement of the ICRP is noteworthy for its warning concerning low IR doses. 

Indeed, the issues of biological effects of low doses of IR are extremely timely, considering not only the long-term effects of past atomic disasters, but also its increasing use and applications in different medical specialties, which have considerably broadened the numbers of exposed subjects. Not surprisingly, there are several studies reporting brain and ophthalmic IR in both patients and interventional radiologists [5,6]. Therefore, the determination of the “real” prevalence and of the biological basis of the brain and ophthalmic long-term effects in of low IR doses is another important problem involving not only radiation medicine and radiobiology, but also other medical branches, such as ophthalmology, neurology and psychiatry. 

Given the scattered information in this field, the aim of this paper was to review the current experimental, epidemiological, and clinical data on the IR-induced cerebro-ophthalmic effects amongst in utero-exposed individuals, children and adults.

The review was carried out according to the PRISMA guidelines [7] by searching PubMed and Google Scholar English papers published from January 2000 to January 2020. The keywords used and combined with “Ionizing radiation” were “Brain”; “Eye”; “Ophthalmic effects”; “Cerebral effects”; “Nuclear disasters”; “Interventional Radiology”. All the authors agreed to include in the review conference abstracts, posters and case reports if published in an indexed journal. The following inclusion criteria were adopted: studies carried out in clinical samples of adults and children/adolescents, and reliable assessment of outcome measures. 

**Figure 1:**
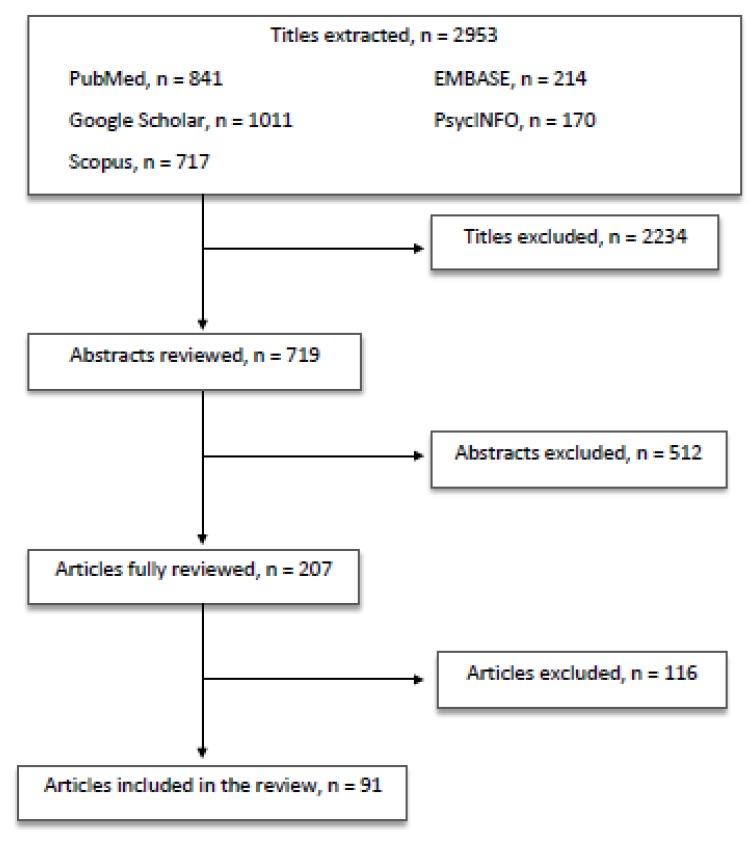
Article selection flow chart.

All the authors equally contributed in identifying potential information specific to this topic among the titles and abstracts of the publications. The first selection excluded 2234 titles because: (a) duplicates arose; (b) they did not concern the scope of the paper; (c) they were not informative enough. The second selection excluded 512 abstracts after being read and reviewed, as the information reported did not fulfill the scope of our paper and/or the presented information did not seem relevant to the discussed topic. Subsequently, 116 articles were excluded after being completely read and evaluated, as they did not provide enough information and/or resulted sufficiently in line with our review. Finally, 91 papers were included in the present review (Figure 1).

The data were discussed and integrated with the findings gathered by the authors after the accident at the Chernobyl Nuclear Power Plant (NPP) and their proposal which they called eye-brain axis.

### 1.1. Ophthalmic Effects in Irradiated Children and Individuals Exposed in Utero

The available literature indicated that subjects exposed prenatally and during childhood to IR are a particular target group with a higher risk for possible acute and long-term IR effects including neurodegenerative diseases. 

For children with brain tumors, craniospinal irradiation poses a significant risk for cataract development for more than two years [8]. It is also well established that repetitive head computerized tomography (CT) scans may enhance risks of IR-induced lens opacification. A study showed that during brain CT scanning, the mean dose for the eye lens was 10.5 ± 3.3, 29.9 ± 8.6 and 34.2 ± 14.9 mGy in children aged 0.8–1 years, 2.0–4.9 years and 5.5–15.5 years, respectively [9]. According to us, such values would require special measures to be taken in order to reduce the possible eye effects. For instance, the combination of topogram-based tube current modulation and barium sulfate or bismuth antimony shields were shown to reduce lens doses by 12.2% and 27.2%, respectively [10]. Even gantry tilting and patient set-up seem to significantly affect eye lens dose [9]. Such simple measures, such as modifying the neck position, shortening the scanning range and reducing the tube potential could decrease the dose to the lens by 89% [11]. 

The results of the so-called “Pittsburgh Project and the Ukrainian-American Chernobyl Ocular Study” (UACOS) showed a significantly higher frequency of posterior subcapsular opacities of the lens following IR, especially in the children irradiated with doses higher than 400 mG [12,13,14]. Data from monitoring children undergoing long-term low-intensity IR exposure in Taiwan confirmed these findings [15]. 

Prolonged (eight years) observation of the eyes among 461 children living in one of the radiation-contaminated areas following the Chernobyl disaster showed soft opacities in the subcapsular layers of the lens, similar to those changes identified in atomic bomb survivors. The opacities were significantly higher in the most exposed than in the less exposed subjects (18.97% vs. 9.3%, *p* < 0.05) [16].

As far as effects of in utero irradiation data, with their unique history of exposure to extensive nuclear testing between 1946 and 1958, the descendants of Marshall Island residents are a typical and dramatic, albeit small, example. The retrospective cohort study of resident women with at least one singleton live birth between 1997 and 2013 in northwest Arkansas using state birth certificate data linked to data from the Arkansas Reproductive Health Monitoring System, a state-wide birth defects registry was performed in order to evaluate the rates of different birth defects. Marshallese infants had higher rates of congenital cataracts (and of truncus arteriosus defect), with Public Risk of 9.3; 95% [17]. 

The problem of intrauterine brain or ocular damage as a result of the Chernobyl disaster is still controversial, although some studies reported that children exposed in utero showed some eye malformations and/or alterations in visual information processing. The most common eye malformations observed were congenital cataracts and retinal angiopathy. The prevalence of congenital cataracts was significantly higher than in the comparison group (2.0% vs. 0.89%), especially if the pregnant mothers received individual total effective doses of 75 mSv or higher (RR = 6.22, 95%) [18,19,20,21,22,23]. Pathological changes of eye vessels at the basis of retinal angiopathy were also widely observed in irradiated vs. the control groups with figures of 176.7‰ ± 15.8‰ vs. 51.98‰ ± 7.81‰. In those subjects irradiated in utero the average thickness of the retina in foveola, as measured by tomography in the long-term period was indeed significantly more robust, than in control subjects (197.75 ± 5.48 μm 181 ± 5.01 μm, *p* < 0.05) [24,25]) [19,23].

The alterations of visual information processing were mainly detected by visual evoked potentials (VEPs). The VEPs to checkerboard reversal pattern took the form of high-amplitude (up to 30.7 μV) biphasic potential with latencies for components P_100_ of 42–152 ms, for N_145_ 75–245 ms and for P_200_ 115–302 ms, respectively (the so-called, “vertex-potential”). Paroxysmal (epileptiform) states represented the clinical equivalents of the pathological “vertex-potential”, perhaps suggesting limbic system irritation. The second feature of VEP was an interhemispheric shift of the maximum of visual information processing from the right, as observed in non-irradiated children (and assumed to be normal) to the left dominant hemisphere. In our opinion, the decreased spectral θ-power, especially in the left fronto-temporal area, as well as the increase in spectral β-power lateralized to the left hemisphere might be also considered as qEEG markers of prenatal irradiation. Taken together, these abnormalities seem to suggest the left hemisphere is more sensitive to in-utero radiation exposure [26,27,28,29,30,31].

### 1.2. Ophthalmic Radiation Effects in Adults

IR-induced (cerebro)-ophthalmic effects in adulthood include cataract, glaucoma, optic neuropathy, retinal angiopathy and dry-eye syndrome.

#### 1.2.1. Cataract

A cataract is an age-related and common opacity of the transparent crystalline. Different types of cataracts are the main cause of blindness worldwide, and the second most common reason for visual impairment after uncorrected refractive errors [32,33]. Cataracts are classified anatomically into nuclear, cortical and posterior subcapsular (PSC) subtypes [34]. The cataract is a well-known IR-induced effect in humans and animals. The PSC subtype is the most common cataract associated with IR exposure [32,33], followed by cortical ones [35]. 

However, there is a limited understanding of the processes leading to cataract formation after IR exposure. It has been proposed that IR provokes damage of germinative zone-dividing cells, at least in the PSCC subtype, resembling a cancer-like pathology of the lens [36]. Further, increased concentrations of reactive oxygen/nitrogen species (ROS and RNS) in human lens epithelial cells have been detected, together with a demonstrated dose-dependent relationship at higher IR doses (>0.5 Gy) [37].

Minor evidence suggests an inverse relationship between lens irradiation dose and cataracts latency [38]. The acute threshold for radiation-induced cataracts of 0.5 Gy was derived from two papers on atomic bomb cataracts assessed after 55–57 years from the exposure: the first paper provided thresholds of 0.6 Gy for cortical cataracts and 0.7 Gy for PSC opacities [39]. The second study reported increased cataract prevalence with a dose of 1 Gy at an odds ratio (OR) of 1.39, as well as a threshold of 0.1 Gy for cataract surgery prevalence, according to the data obtained from 3761 atomic bomb survivors [40].

**Figure 2:**
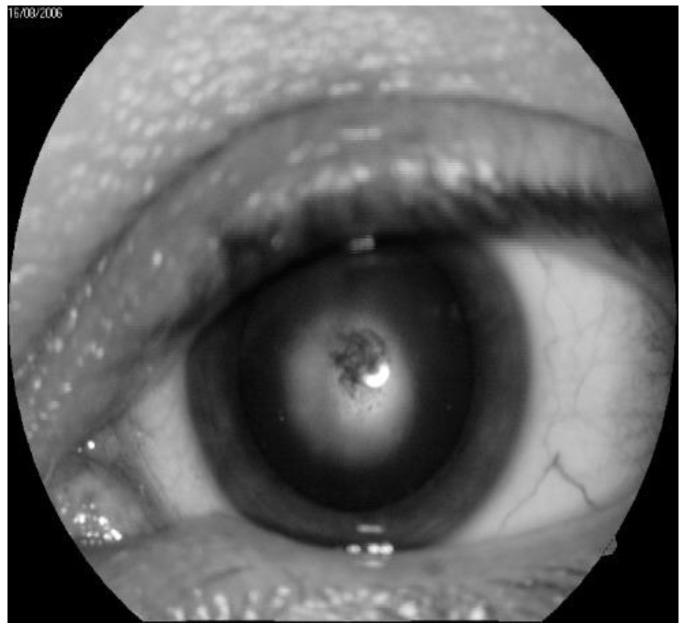
Typical example of radiation cataract at the second stage. (by courtesy of P. Fedirko and T. Babenko from the National Research Center for Radiation Medicine of the National Academy of Medical Sciences of Ukraine (NRCRM), Kyiv, Ukraine, copyright of co-authors [42].

In the immediate aftermath of the Chernobyl disaster, 226 cases of specific IR cataracts were recorded, while 179 cases were observed in the long term. The greatest number of cases of cataracts was diagnosed eight/nine years after IR exposure, but new cases continue to be detected one even after 29 years following radiation exposure [41] (a typical example is depicted in Figure 2). In agreement with the literature, we established that the typical clinical picture of radiation cataract may arise at doses considerably lower than 0.25 Gy, with the threshold for cortical or PSC cataracts being of 0.34–0.5 Gy [41,42,43]. In addition, a complete ophthalmologic examination of 53 acute radiation sickness (ARS) convalescents exposed to high IR doses demonstrated that 39 showed a combination of involutional and radiation cataracts.

In any case, currently, there is still much uncertainty and controversy regarding the relationship among cataract development, dose protraction and latency period, as well as the stochastic versus deterministic nature of IR-induced cataracts [44]. Therefore, a long-term monitoring of irradiated patients with cataracts, as well as a thorough analysis of different factors acting on the lens, are strongly needed in order to determine which class of phenomena the radiation cataracts can be attributed to.

#### 1.2.2. Glaucoma

A meshwork of cells lying at the junction of the iris and the cornea is responsible for aqueous drainage. Failure of this drainage causes glaucoma, a disorder in which high levels of intraocular pressure (IOP) reduce the blood supply to the eye and eventually destruct the structures in and around the optic nerve that causes clinically significant visual dysfunctions, and eventually blindness [45]. 

The possible relationship between IR and glaucoma was first reported among Japanese atomic bomb survivors, at a percentage of around 10% [46,47].

Our data showed that Chernobyl clean-up workers have a significantly higher risk of involutive changes of the anterior chamber angle (ACA) (trabecular zone sclerosis, pigment deposition and exfoliative particles in it, narrowing of the venous sinus of the sclera, as well as the tendency to ACA constriction in some areas), as compared with the control group (RR = 3.5, 1.27–9.5, χ² = 7.48, *p* = 0.031). According to us, such early onset of ACA morphological changes may lead to an increased incidence of open-angle glaucoma in the long-term period [48]. In a cohort of the liquidators (4017 people) during 13 years of dynamic monitoring (18 years after the Chernobyl accident), glaucoma was diagnosed in 28 patients (6.97 per 1000 persons of the group monitored): the annual incidence was, thus, 0.54 per 1000 persons, with a growth of incidence as the IR dose increases to 0.25 Gy [49].

The prevalence of glaucoma among 449 inhabitants of zones of tightened radio environmental control was 6.68 per 1000 people, higher than that in the Kyiv region (4.12 per 1000 people) [50]. Further, when re-examining 434 persons who had been working at transforming the NPP into an ecologically safe system in 2009–2011, the primary incidence of glaucoma was shown to be 13.82 per 1000 people, significantly higher than that of all Ukrainian population (0.65 per 1000 people, or 0.07%) [50].

Today, the use for medical purposes of X-ray represents the most frequent way of exposure to IR for the human brain and eye. According to different studies, the percentage of vascular glaucoma in individuals exposed to high dose-rate radiotherapy to the eye ranges between 7% and 48% [51,52,53,54,55]. These findings, suggesting the contribution of possible IR-induced small ocular vessels to the development of post-radiation normal-tension glaucoma (NTG), are strongly consistent with epidemiological data which define radiation risks for cerebrovascular pathology at doses >0.1 Gy [56], >0.15 Gy [57] and >0.25 Gy [58]. Interestingly, currently, some authors consider glaucoma a sort of neurodegenerative disease, as they postulate that there might exist a brain component that is independent from the eye damage and that would play an etiological role in its development [59]. Diffusion tensor imaging (DTI) indices, specifically in the optic tracts, optic nerves and optic radiations, result in changes in patients with primary open-angle glaucoma (POAG). POAG causes, or is associated with, microstructural changes (decrease in fiber numbers, FN) involving brain regions associated with vision (BA19), depression (BA10/BA46/BA25) and memory (BA29), which supports the concept that POAG may affect neuroanatomical connections in the human brain not only within, but also beyond the visual pathways [60]. Similar findings were reported in normal-tension glaucoma (NTG), as DTI detected white matter damage (WM) in the four regions associated with visual and visual-related functions, specifically in bilateral posterior thalamus, bilateral sagittal striatum, bilateral cingulum hippocampus and bilateral fornix/stria terminalis. Moreover, DTI parameters in those brain areas turned out to correlate with such specific glaucoma indices, such as the mean deviation of the visual field and retinal nerve fiber layer thickness, and occurred before detectable visual field loss [61]. The primary visual cortex also exhibited more severe functional deficits than higher-order visual brain areas in glaucoma [62]. It was hypothesized, therefore, that glaucoma deterioration is already present in the eye and the brain before substantial vision loss can be detected clinically using current testing methods. In Japanese NTG patients, DTI revealed white matter degeneration even in the corpus callosum: this would suggest the presence of neurodegeneration that cannot be explained on the basis of propagated retinal and pre-geniculate damage [59]. In glaucoma, which is typically not considered a demyelinating disease, there was an increment in radial diffusivity within the optic radiations, which was confirmed by the topographically linked delay of visual evoked potential latency, a functional measure of demyelination [63]. Demyelination is believed to be the biological marker of the delayed radiation therapy damage, which typically begins six months and later after finishing the treatment course, is characterized by steady cognitive impairment and radiographically visible neuropathological changes, and it is considered to be irreversible and progressive [64,65]. Some reports mentioned the disruption of two measures of semantic memory, namely the postencoding retrieval from long-term memory of words auditorily presented and recognition of a large set of nameable pictures, indicating an impairment of higher perception and memory in auditory and visual modalities. A radiation therapy-related disruption of glial mitosis, especially of oligodendrocytes, may lead to temporary demyelination, and has been described to account for the already-mentioned neurocognitive deficits [66,67,68]. Interestingly, an increased incidence of multiple sclerosis, a genuine demyelinating disease, was described in 2005–2010 in the North-West Ukraine regions, mainly affected by the Chernobyl accident fallouts. Specifically, the highest level was revealed in Western (71.8 per 100,000) and central (59.0 per 100,000) areas of Ukraine in comparison with 18.0–44.0 per 100,000 in South-Eastern areas. It is reasonable that exposure to radionuclides accounts for a higher incidence of multiple sclerosis in these most affected areas after the disaster, and that demyelinating and degenerative processes in the brain and eye structures may result from or be a long-term consequence of IR [69].

### 1.3. Optic Neuropathy, Angiopathy and Chorioretinal Dystrophies

#### 1.3.1. Optic Neuropathy

Radiation-induced optic neuropathy (RION) is a form of delayed radionecrosis of the anterior visual pathways, which develops within months to years after external cranial irradiation, and provokes severe and irreversible vision loss. High-resolution MRI of the optic nerves usually demonstrates enhancement of a discrete segment of the intracranial prechiasmatic optic nerve, often with accompanying expansion and T2 hyperintensity [70]. In a retrospective study comparing RION cases with matched control subjects, 13 RION patients (for a total of 18 eyes) had received doses below published “safe” thresholds (<55 Gy; <8–10 Gy for stereotactic radiosurgery) [71]. Similarly, a case of optic neuropathy and retinopathy was reported following IR dose traditionally thought to be safe in a 44-year-old female patient after receiving proton beam radiotherapy (20 Gy dose delivered in two 10 Gy fractions) on the left eye for a uveal metastasis of lung cancer [72]. 

#### 1.3.2. Angiopathy and Angiosclerosis of the Retina

Angiopathy of the retina is the eye pathology emerging several years following the IR exposure. The age of the irradiated individuals and the time of staying under risk are the major risk of retinal angiopathy, while the contribution of IR dose is smaller [24,73]. 

**Figure 3:**
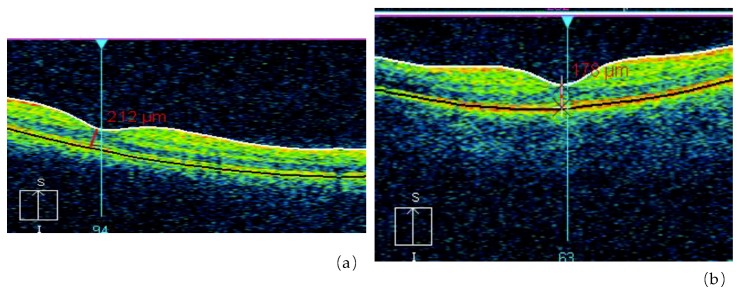
Retinal abnormalities in the irradiated group detected with Optical Coherence Tomography (OCT) (**a**), as compared with non-exposed control subjects (**b**) (by courtesy of P. Fedirko and T. Babenko from the National Research Center for Radiation Medicine of the National Academy of Medical Sciences of Ukraine (NRCRM) Kyiv, Ukraine, copyright of co-authors [16,20].

**Figure 4:**
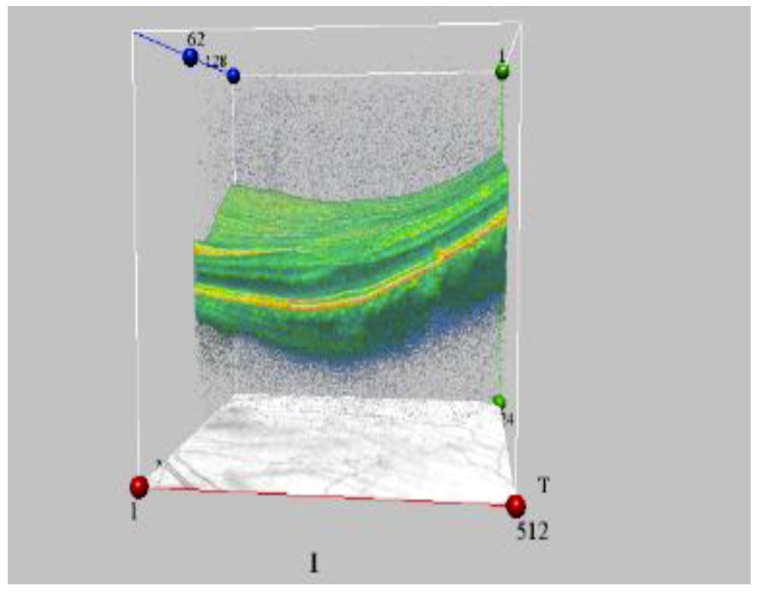
The initial stage of macular degeneration in ARS 1st grade convalescent (OCT data) (by courtesy of P. Fedirko and T. Babenko from the National Research Center for Radiation Medicine of the National Academy of Medical Sciences of Ukraine (NRCRM), Kyiv, Ukraine, (copyright of co-authors [75,76]).

One of our studies revealed a significant increase of retinal angiopathy and angiosclerosis in a cohort of liquidators of the Chernobyl NPP accident (314.8 ± 14.5 per 1000 people in 1993 and 911.9 ± 19.7 per 1000 people in 2004). The relative risk of angiopathy, in comparison with a control group, was 2.6 for a dose up to 0.05 Gy, 2.75 for doses ranging from 0.05 to 0.099 Gy, 2.86 for doses between 0.1 and 0.249 Gy and 2.93 for a dose of 0.25 Gy or higher. In several liquidators who were initially diagnosed with angiopathy and followed up, a transformation of angiopathy into angiosclerosis was noted: walls of arteries became thicker, the lumen of vessels decreased and the caliber became uneven (Figure 3 and Figure 4). That is why the prevalence of the retina angiosclerosis increased considerably by time, mainly in relatively young age groups [24,73,74].

#### 1.3.3. Chorioretinal Dystrophies

The most common kind of retinal pathology in irradiated people is central chorioretinal dystrophy (macular). The data of cohort research and mathematical modeling made it possible to establish that the risk of macular dystrophy mainly depends on the age of irradiated people at the moment of examination, staying under risk for a long time and IR dose [41,73,75]. In one of our studies of an optical coherence tomography of the macular zone in convalescents of acute radiation sickness in the long-term period (25 years following the Chernobyl accident), the subjects with acute radiation sickness were divided into two subgroups: (1) patients with the established diagnosis of macular retinal degenerations; (2) patients with no pathological changes in the macular zone. The results showed that the general architecture of the retina of the two subgroups was similar, as they both presented a statistically reliable increase in retinal thickness in the foveola, in comparison with non-irradiated control subjects [75,76]. We also considered that change of retinal thickness in the macular zone could be the cause of metamorphopsia development (a curvature of a form and the sizes of objects).

### 1.4. Dry-Eye Syndrome

Dry-eye syndrome is another possible cerebro-ophthalmic effect that may develop in patients who receive whole-brain radiotherapy as late toxicity, although in general practice, dose to the lacrimal gland is not constrained (maximum constraint <40 Gy), although it would require to be taken care in order to prevent it [77]. In the study of 213 meningioma patients receiving radiotherapy between 2000 and 2013, 15 dry-eye (7%) cases were found at a median dose to affected lachrymal glands of 1.47 Gy and a median dose to affected lenses of 1.05 Gy [78]. 

To summarize, although meager, available data suggest that IR is an essential factor able to trigger damage and degeneration of different eye structures, as well as of visual pathways in the CNS, while impairing significantly not only visual function per se, but also a higher level of visual and perhaps other information processes [79].

### 1.5. Ophthalmic IR Effects in Interventional Radiology

In the last decades, the worldwide development of interventional radiology was great, providing significant human health benefits, but also increasing the radiation exposure in the patients and in the health workers, up to become the largest artificial source of IR [80,81,82]. Nowadays, it is applied by several medical figures, including cardiologists, vascular surgeons, neuroradiologists, orthopedists and urologists, since it includes all activities using radiological or radionuclide devices for diagnostic and therapeutic purposes [81]. As already mentioned, although eyes and the brain are the most vulnerable organs, the protection provided is generally insufficient, as the protection of the ocular lenses by leaded glasses may be incomplete and that of the brain by radio-absorbent surgical cap minimal [83,84]. As a result, the eye-damaging effects of IR are one of the main health problems in interventional radiology. The most radiosensitive ocular component is the lens: therefore, the most common consequence of eyes IR exposure in interventional radiology operators is the lens opacity, which can progress up to cataracts [79,85,86]. Several data demonstrated a higher frequency of early lens opacity and cataracts in these workers [87,88,89,90], especially among interventional cardiology [88,91,92,93,94]. A recent study indicated that around 25% of interventional cardiologists might be at risk of developing an early radio-induced cataract [88]. Although in the early stages of lens opacity the vision may be intact, such condition tends to worsen progressively with the increase of the doses and the time exposure, up to causing impaired vision, which requires surgery [79,85,86]. Thus, radio-induced lens alterations should not be undervalued, even because they are associated with a severe impact on professional proficiency, quality of life and career span [3,79,85,86,95,96,97,98,99].

In conclusion, it would be necessary to expand research studies on eyes and brain consequences of interventional radiology, as well as on the radioprotection devices and safety procedures aiming to contain these undesirable side effects. Indeed, although technology has provided major protective benefits both to physicians (and patients), currently radioprotection protocols are still ineffective and poorly uniform [83,85,86]. For this purpose, new predictive models of computational dosimetry could be useful and implemented [100]. Furthermore, it would also be important to provide training courses on radioprotection and workers’ safety for all interventional radiology workers, since they are often lacking and not standardized [83,86].

## 2. Discussion

The data deriving from the current literature review, as well as from the findings gathered from our personal experience facing the consequences of the Chernobyl NPP disaster, support the notion of the peculiar radiosensitivity of the eye. We would like herein to propose our concept that ophthalmic damage would be just a marker of more generalized brain damage, on the basis of what we called "eye-brain axis”, as supported by evidence that the eye, specifically the retina, is just a brain expansion. We also propose that the study of the human visual pathway might play an important role in investigating the CNS in both physiological and pathological conditions [63].

The pupillary light reflex (PLR), a mechanism for light adaptation, is an evident example of the connections between eyes and brain. Indeed, although PLR is often described as an immutable reflex, it can be modulated by cognitive factors. In rhesus macaques, the microstimulation of the frontal eye field in the prefrontal cortex was shown to modulate the gain of PLR, while adding complexity and flexibility to a basic brainstem circuit [101]. In laboratory animals undergoing light deprivation (LD), peculiar morphological changes of retinal ganglion cells (RGCs) [102] and layer five of pyramidal cells of the primary visual [102], auditory [102] and motor cortex areas [103] were reported [102,103]. At the same time, LD also caused a depressive phenotype [102,103]. Interestingly, depressive syndromes are also one of the most frequent long-term effects of atomic bombings, nuclear testing and radiation accidents [104,105]. Incidentally, it is noteworthy that in exposed subjects developing affective and cognitive disorders, we could detect an association between IR and polymorphisms of the serotonin transporter gene SLC6A4, being serotonin, the main neurotransmitter involved in depression [105].

Different data support the notion of our proposal of the eye-brain axis, and how the onset of degenerative processes in one may influence those in the other. Elderly individuals without dementia showed thicker retinal nerve fiber layer (RNFL) correlating with better MRI variables in visual pathway regions and also in areas typically affected by Alzheimer’s disease (AD) neurodegenerative processes [106]. Furthermore, some evidence suggested that in healthy aging the thinner macular ganglion cell-inner plexiform layer might be associated with a lower gray matter volume [107]. Some CNS neurodegenerative conditions can be accompanied by architectural and electro-physiological abnormalities of the retina, hence supporting the usefulness of eye examination tools to early detection of the neurodegenerative processes, such as in AD where visual deficits are common. By using diffusion tensor imaging (DTI), a study demonstrated optic nerve WM alterations in AD, as compared with control subjects [108]. Moreover, in posterior cortical atrophy, a variant of AD characterized by a high-level of visual deficits like alexia and agnosia, structural MRI analysis indicated a great loss of gray matter in the occipital and parietal cortices, lateralized to the hemisphere contralateral to the visual loss [109]. 

The reverse pattern is also consistent within the frame of hypothetical the eye-brain axis. In subjects affected by age-related macular degeneration, retinal damage seems to be the main cause of the brain WM degeneration that was observed in this condition. In particular, abnormalities were reported in WM fascicles projecting to the primary visual cortex corresponding to the area of retinal damage (fovea), with their magnitude being correlated with visual acuity loss [110]. In subjects with congenital aniridia, the WM structure appeared altered not only in visual tracts, but also in different brain structures belonging to the posterior visual pathways, such as bilateral optic tract, bilateral optic radiation, forceps major and bilateral superior longitudinal fasciculus, as well as right posterior corona radiata [111]. Further, degenerative processes were described not only in the retina, but also in the brain optic radiation in retinitis pigmentosa (RP) [112].

After irradiation of retina cell culture in vitro at doses ranging from 1 up to 2 Gy, which is the incidental dose received by the healthy retina per fraction when the standard treatment is delivered to the brain, at day two, an evident loss of cell viability and βIII-tubulin immunostaining was evident, while highlighting marked neuritic damage [2]. In eight patients who developed retinopathy following plaque radiation treatment for choroidal melanoma, the average total retinal blood flow (TRBF) was significantly lower, and retinal blood oxygen saturation indications were higher in the retinopathy eye, compared with the other, which indicates alterations of retinal vascularization, similar to a rapidly developing diabetic retinopathy [113]. 

Therefore, IR should be considered as an essential factor triggering damage and degeneration in various eye structures and associated or not brain areas. Moreover, such radiation-induced pathophysiological alterations may lead to secondary cognitive/neuropsychiatric effects, whose thorough discussion is beyond the scope of the present paper. In any case, the study of human visual pathway plays an important role in investigating neurodegeneration in CNS due to its unique hierarchical architecture allowing tracing trans-synaptic degeneration, which is unlikely to be possible in a whole-brain connectivity model [63]

## 3. Conclusions

The bulk of available information strongly indicates that the eye and brain are extremely radiosensitive and radiovunerable organs. It is now evident that detrimental effects may occur at their levels even at low and/or chronic IR doses. This is a recent concept eliciting several issues that strongly challenge current research, clinical practice and safety for several medical workers, and even raising novel questions concerning future space exploration. Indeed, cognitive and visual disturbances, mainly retinal phosphenes, have been reported during space missions [114]. Retinal phosphenes during space travel can alter perception, as the light is visible where there is no light and are extremely dangerous in conditions requiring reliable processing of visual information [115,116]. Retinal phosphenes may induce overproduction of free radicals and great retinal lipid peroxidation, with a consequent strong biophoton emission, which can really be perceived and interpreted by the brain as bright flashes [115,117]. It was speculated that the effects of this type might also affect other areas of the brain sensory system, as well as brain regions responsible for cognitive functions, hence providing another support to our notion of the eye-brain axis [118]. 

In conclusion, according to available information that eye alterations may induce or may be associated with brain dysfunctions and vice versa, we propose that a deepening and more extensive use of diagnosis of eye pathologies might represent early and easily obtainable markers of possible low dose IR-induced brain damage.

Future studies of possible brain and ophthalmic IR effects in humans should be focused on the search for specific morphological, visual, neurophysiological and neuropsychological markers of higher visual perception processing disruption in a broader sense. Additional research is still needed in the following areas: a comprehensive evaluation of the overall effects of IR on the eye, dosimetry methodology and dose-sparing optimization techniques, additional high-quality epidemiology studies and a basic understanding of the mechanisms leading to different eye disorders and to their interactions with brain processes. It is also essential to implement follow-up studies on medical and biophysical monitoring of various cohorts involved in radiation-related activities in different contexts (atom industry workers, clean-up workers, persons irradiated in utero, interventional radiologists, servicemen, astronauts, and so on).
